# Listeria Bacteremia With Septic Brachial Artery Thrombosis and Pacemaker Infection in an Elderly Woman With a Prosthetic Aortic Valve and Recurrent Pacemaker Implantation

**DOI:** 10.7759/cureus.87139

**Published:** 2025-07-01

**Authors:** Abbas Merchant, Haashim Rahman, Aaryan Patel, Adithya Nagendran, Anushree V Murthy, Umar Hanif, Rida Merchant, Hira Rahman, Ishan Deshmukh, Constantino G Lambroussis

**Affiliations:** 1 Anesthesiology, Lake Erie College of Osteopathic Medicine, Erie, USA; 2 Physical Medicine and Rehabilitation, Lake Erie College of Osteopathic Medicine, Erie, USA; 3 Internal Medicine, Rochester Regional Health, Rochester, USA; 4 Internal Medicine, Saint James School of Medicine, Arnos Vale, VCT; 5 Psychiatry, Albany Medical College, Albany, USA; 6 Internal Medicine, St. George's University School of Medicine, St. George's, GRD; 7 Vascular Surgery, Lake Erie College of Osteopathic Medicine, Elmira, USA; 8 Osteopathic Medicine/Family Medicine, Lake Erie College of Osteopathic Medicine, Elmira, USA

**Keywords:** bacteremia, brachial artery thrombosis, cardiac device-related infection, immunocompromised hosts, infective endocarditis, listeria monocytogens, pacemaker infection, prosthetic valve infection, septic thrombosis, tavr (transcatheter aortic valve replacement)

## Abstract

A 77-year-old female with a history of coronary artery disease status post-coronary artery bypass grafting, aortic stenosis treated with transcatheter aortic valve replacement, and recent dual-chamber pacemaker implantation for high-grade atrioventricular block presented with recurrent syncope, right brachial artery thrombosis, and persistent bacteremia. Blood and thrombus cultures grew *Listeria monocytogenes*, leading to pacemaker extraction and a prolonged course of antibiotic therapy. This case illustrates the diagnostic challenges associated with systemic *Listeria* infections in patients with prosthetic devices and emphasizes the importance of early blood culture collection in patients with unexplained clinical symptoms.

## Introduction

*Listeria monocytogenes* is a gram-positive, facultatively anaerobic bacterium primarily transmitted through contaminated food. It poses significant health risks, especially to immunocompromised individuals, including the elderly, pregnant women, and neonates. The organism can persist on inadequately sanitized food processing equipment, leading to contamination of ready-to-eat meats, unpasteurized dairy products, soft cheeses, and raw or undercooked vegetables [1]. Its capacity to evade immune responses and replicate intracellularly allows for systemic dissemination, potentially resulting in severe infections such as sepsis, meningitis, and encephalitis [2]. Although *Listeria* infections most commonly present as gastroenteritis or central nervous system disease, vascular complications like arterial thrombosis are exceedingly rare and remain poorly documented in the literature. The prevalence of *Listeria* infection varies among populations and is shaped by social determinants of health, including poverty and limited access to safe food sources, which influence both exposure risk and clinical outcomes. Socioeconomic disparities have been linked to higher rates of *Listeria* infection in marginalized groups, highlighting the importance of addressing these factors in both patient care and public health strategies [3].

## Case presentation

This case details the complex medical course of a 77-year-old woman with multiple cardiovascular conditions, including coronary artery disease (CAD) status post-coronary artery bypass grafting (CABG), aortic stenosis treated with transcatheter aortic valve replacement (TAVR), and recent dual-chamber pacemaker placement for high-grade atrioventricular (AV) block. Vital signs on presentation showed a blood pressure of 176/76 mmHg, a heart rate of 58 bpm, a respiratory rate of 18, an oral temperature of 36.2°C (97.2°F), and an oxygen saturation of 94%. Her condition became further complicated by recurrent hospitalizations, ultimately leading to the identification of *L. monocytogenes *bacteremia and right brachial artery embolism, which necessitated pacemaker extraction due to persistent infection.

She was initially hospitalized for severe nausea, vomiting, and abdominal pain. A CT scan confirmed a small bowel obstruction, which was managed conservatively with bowel rest and nasogastric decompression. She improved without requiring surgery and was discharged after a few days. No blood cultures were taken at that time, and she exhibited no systemic signs of infection. However, it is possible that these gastrointestinal symptoms represented an early manifestation of *Listeria *infection that had not yet become clinically apparent.

**Figure 1:**
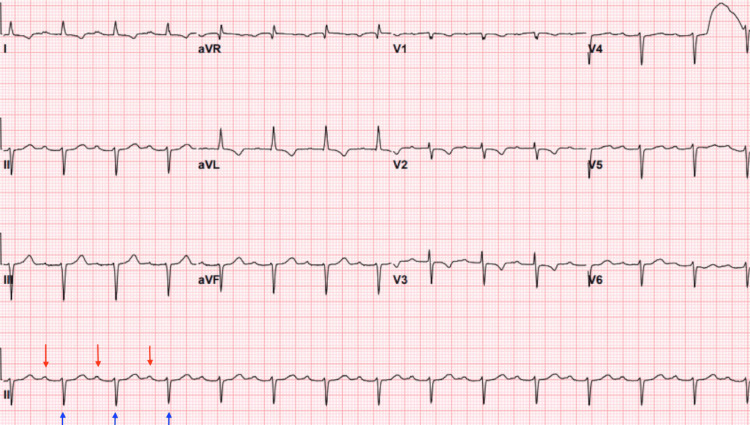
Electrocardiogram The electrocardiogram revealed sinus rhythm with markedly prolonged PR intervals. Telemetry monitoring captured intermittent episodes of complete (third-degree) AV block, characterized by AV dissociation with independent atrial activity (red arrow) and ventricular activity (blue arrow), along with periods of ventricular escape rhythm. These findings were accompanied by significant pauses, consistent with advanced AV nodal disease and high-grade conduction abnormality. AV, atrioventricular

Approximately one month later, she presented again with multiple episodes of syncope and unresponsiveness. An electrocardiogram revealed a significantly prolonged PR interval, and telemetry captured intermittent third-degree AV block with significant pauses (Figure 1). The cardiology and electrophysiology teams were consulted, and she was initially managed with temporary transvenous pacing. Given the findings, a permanent dual-chamber pacemaker was successfully implanted, and she was discharged in stable condition. At that time, there were no signs of systemic infection. However, *L. monocytogenes *has been reported in rare cases to cause high-grade AV block, raising the possibility that the infection may have contributed to her initial presentation.

**Figure 2:**
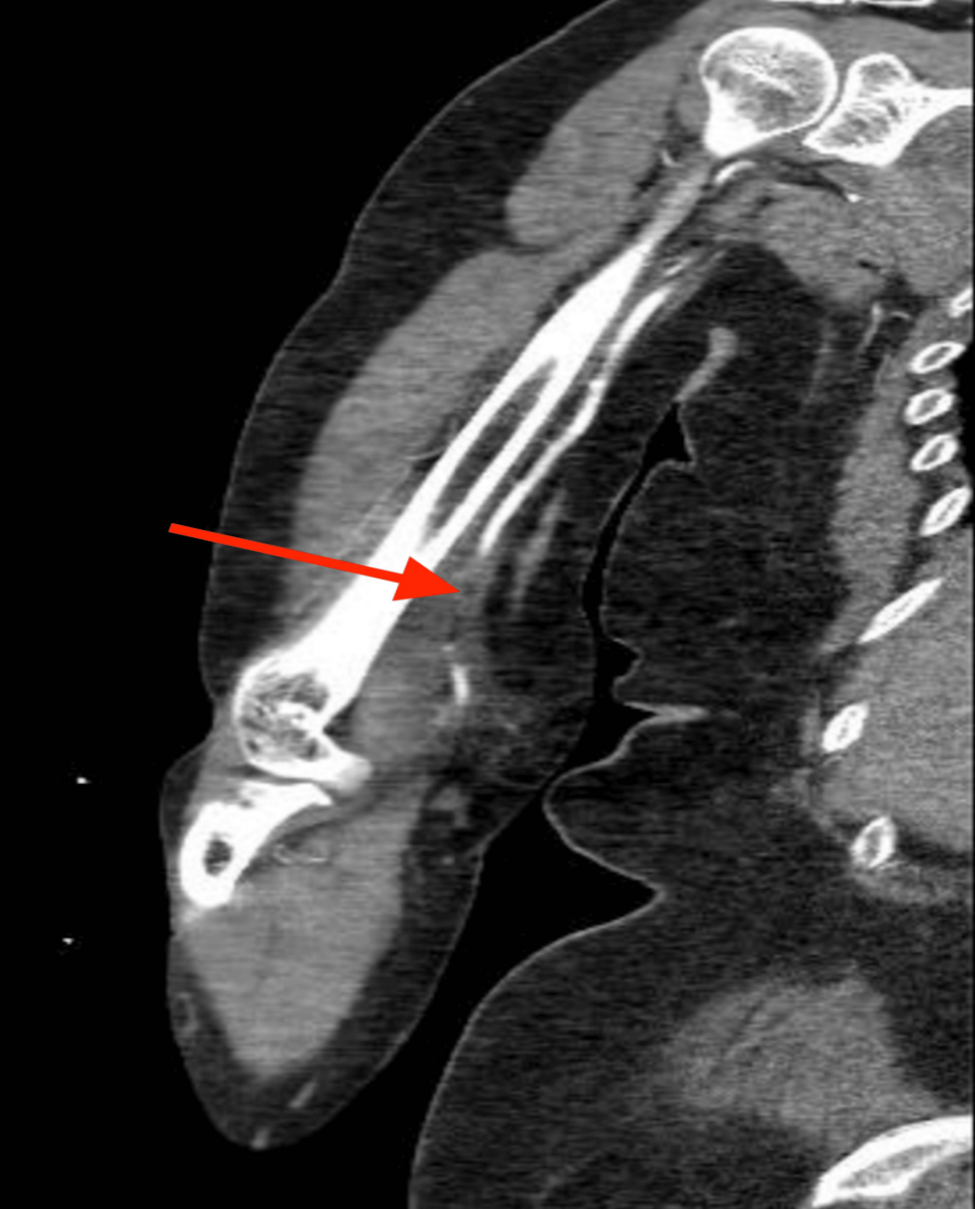
CT angiogram CT angiogram of the right upper extremity demonstrated an abrupt contrast cutoff within the brachial artery, consistent with acute arterial occlusion. There was no visualization of distal reconstitution, indicating impaired perfusion beyond the site of occlusion.

**Figure 3:**
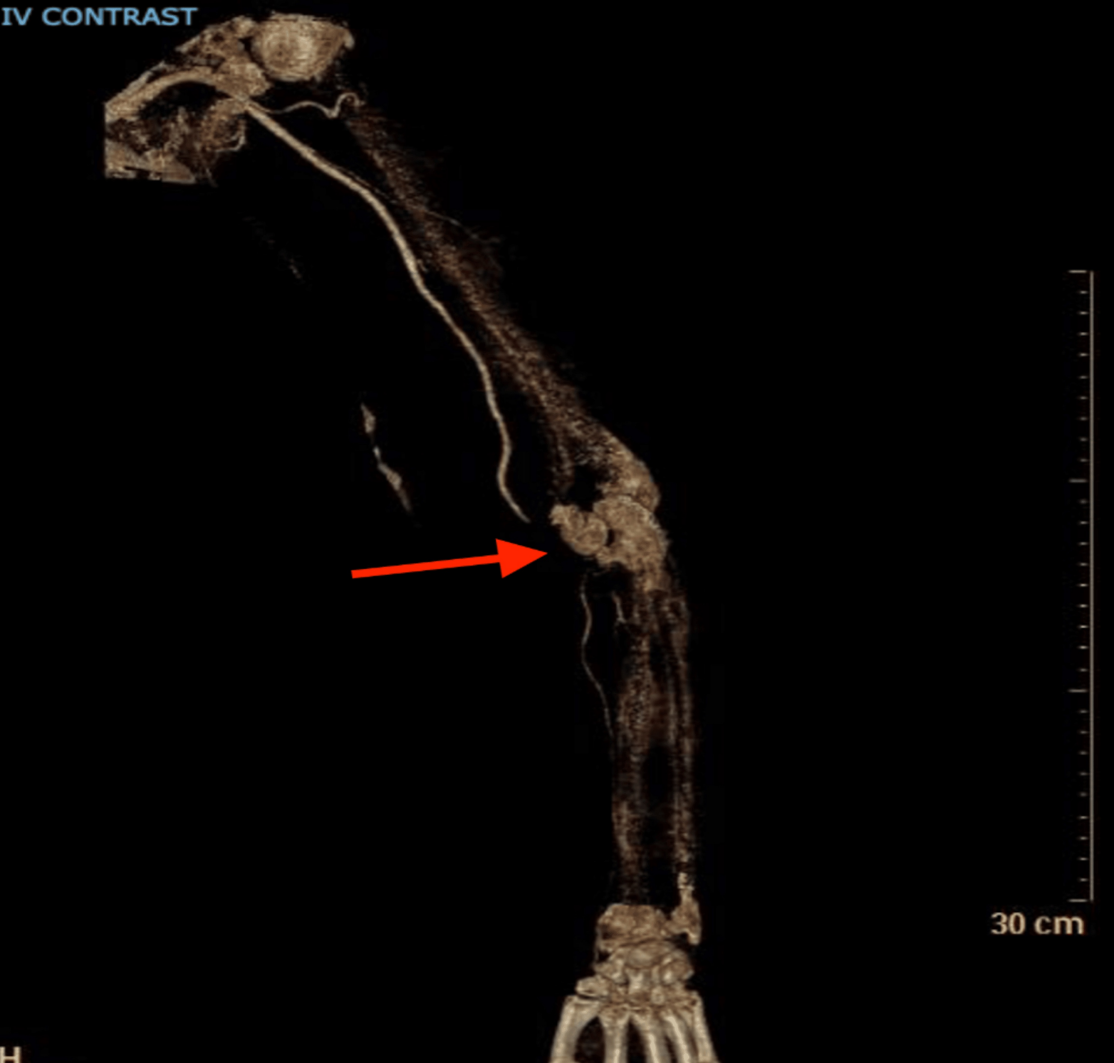
3D reconstruction from a contrast-enhanced CT angiogram The 3D reconstruction of the upper extremity from a contrast-enhanced CT angiogram reveals acute thrombosis of the brachial artery. There is an abrupt cutoff of contrast flow in the mid-to-distal brachial artery (red arrow), with absent distal opacification, consistent with complete occlusion. The affected vessel appears irregular, and there is no evidence of contrast extravasation. These findings are indicative of acute limb ischemia due to arterial thrombotic occlusion.

A few weeks later, the patient returned with acute right upper extremity pain. She described worsening, stabbing pain localized to the antecubital fossa and radiating to her thumb. On examination, pulses were diminished, although there was no associated weakness or numbness. Laboratory evaluation revealed leukocytosis. CT angiography confirmed occlusion of the right brachial artery (Figure 2). To further delineate the extent and anatomical details of the occlusion and to assist with preprocedural planning, a 3D reconstruction of the CT angiogram was performed (Figure 3).

Vascular surgery was consulted, and the patient was started on a heparin drip. Blood cultures were obtained and later grew L. monocytogenes in multiple bottles, raising concerns for septic emboli. She was initiated on intravenous antibiotics, including ampicillin and gentamicin. A right brachial artery embolectomy was performed, and cultures from the extracted thrombus also grew *Listeria*.

**Figure 4:**
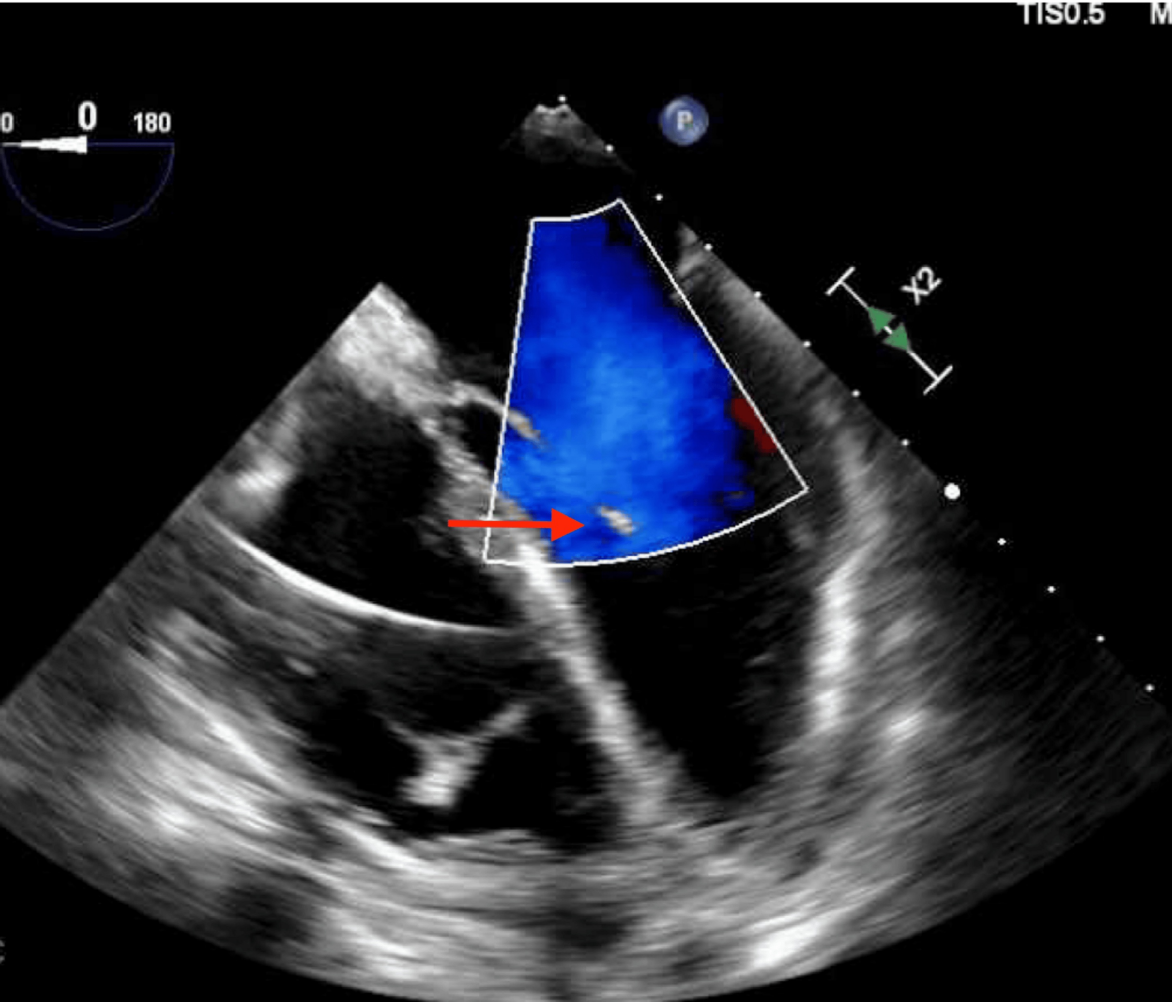
Echocardiogram The RV was normal in size, with a basal diameter of 4.0 cm (normal <4.2 cm) and preserved systolic function. A pacing lead was visualized in the RV. A very small mobile density, measuring approximately 2 mm, was seen attached to the RV endocardium adjacent to the lead. This finding is more likely a fragment of RV myocardium than a vegetation. RV, right ventricle

**Figure 5:**
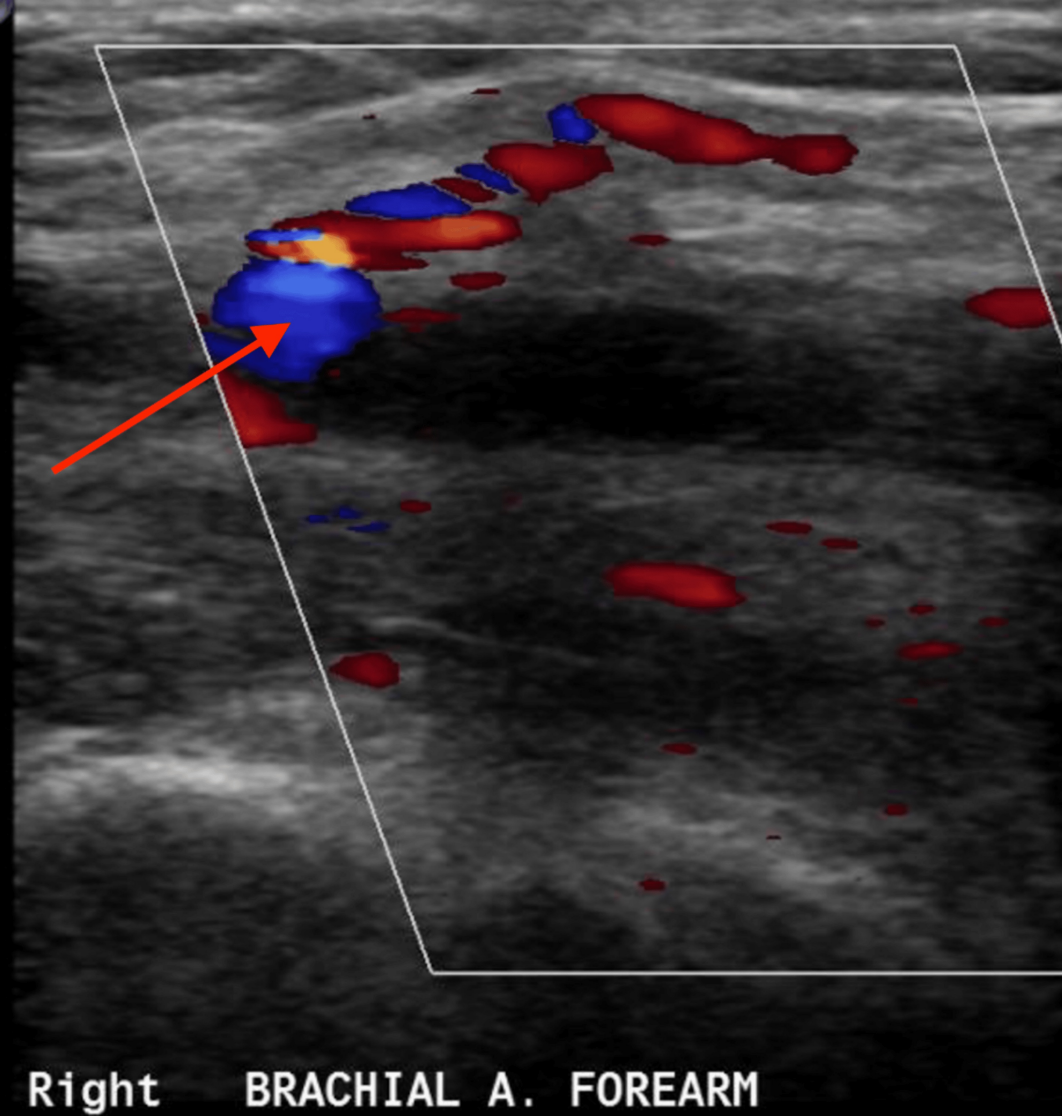
Color Doppler ultrasound Color Doppler ultrasound of the right brachial artery revealed a thrombus, seen as a round anechoic structure exhibiting bidirectional flow on Doppler imaging, producing the classic “yin-yang” sign. The lesion was located adjacent to the brachial artery in the forearm, consistent with turbulent flow within a contained arterial wall defect. These findings are suggestive of a brachial artery thrombus, likely secondary to prior trauma or vascular intervention.

Despite appropriate antibiotic therapy, her blood cultures remained persistently positive. A transthoracic echocardiogram did not reveal any vegetations; however, a transesophageal echocardiogram (TEE) identified a small, mobile density near the pacemaker lead in the right ventricle, raising suspicion for a pacemaker-associated infection (Figure 4). The persistence of *Listeria* bacteremia in the context of arterial thrombosis heightened concern for a deeper or disseminated source of infection. Given her recent pacemaker implantation, a device-related infection was strongly considered. To further evaluate the etiology of the right upper extremity ischemia, a color Doppler ultrasound of the right brachial artery was performed. This revealed an intraluminal thrombus, visualized as a round, anechoic structure with bidirectional swirling flow on Doppler imaging, producing the characteristic “yin-yang” sign, suggestive of a partially occlusive thrombus or pseudoaneurysm (Figure 5).

The presence of *Listeria*-infected thrombus in the brachial artery suggested widespread dissemination, likely originating from the gastrointestinal tract or prosthetic cardiac devices. In patients with prosthetic materials, *Listeria* can invade the bloodstream, seed thrombi, and lead to systemic infection. Given the persistent bacteremia despite appropriate antibiotic therapy, the pacemaker was extracted. Although cultures from the explanted pacemaker leads were negative, the high risk of endovascular infection and the patient’s clinical course justified device removal.

Following pacemaker extraction, the patient developed purulent drainage from the right forearm embolectomy site, raising concerns for a soft tissue infection. Ultrasound imaging revealed a fluid collection, suspected to be a seroma, hematoma, or abscess. The patient continued her course of antibiotics, and the forearm was managed conservatively with close monitoring.

She completed two weeks of gentamicin and was scheduled to complete a six-week course of ampicillin. Weekly blood tests, including complete blood count and basic metabolic panel, were performed to monitor for recurrent infection and potential nephrotoxicity from gentamicin.

Throughout hospitalization, she remained hemodynamically stable without recurrence of AV block. The electrophysiology team recommended outpatient cardiac monitoring for four weeks while she completed antibiotic therapy. Although she remained electrically stable, discussions regarding future pacemaker reimplantation were initiated. Given the ongoing risk of infection, reimplantation was deferred until infection clearance was confirmed. A leadless pacemaker (Micra) was considered to reduce the risk of future infections.

The interdisciplinary approach, featuring timely pacemaker extraction and targeted antibiotic therapy, was critical in stabilizing her condition. Continued follow-up was deemed essential to ensure complete eradication of the *Listeria* infection and to assess the need for future pacemaker reimplantation.

## Discussion

This case highlights the key challenges in managing L. monocytogenes infections, particularly in patients with cardiovascular comorbidities and implanted prosthetic devices. While *Listeria i*nfections typically present as meningitis, sepsis, or gastroenteritis, this case illustrates a rare complication - septic embolization leading to brachial artery thrombosis. Persistent bacteremia despite appropriate antibiotic therapy underscores the difficulty of eradicating infections associated with implanted medical devices, especially in patients with CAD, prior CABG, TAVR, and recent pacemaker implantation, all of which increase susceptibility to endovascular infections.

The decision to remove the pacemaker was driven by continued positive blood cultures, raising strong suspicion for a biofilm-associated infection resistant to antibiotics alone. Although cultures from the extracted pacemaker leads were negative, the clinical suspicion for device-related infection remained high. Managing *Listeria *infections in patients with prosthetic material is particularly challenging and often requires prolonged intravenous antibiotic therapy, typically ampicillin combined with an aminoglycoside for synergy. Complete hardware removal is generally recommended in confirmed cases to prevent relapse [4]. The timing of pacemaker reimplantation must be carefully considered to avoid reinfection, and leadless pacemakers such as the Micra device may reduce the risk of future device-related infections.

Septic emboli due to *Listeria *are uncommon and typically affect the central nervous system or large arteries. In this case, embolization involved a peripheral artery, resulting in critical limb ischemia [5]. This underscores the need to consider septic embolization in patients with persistent *Listeria *bacteremia and vascular complications, especially in those with prosthetic devices and complex cardiovascular histories. Clinicians should also include marantic (non-bacterial thrombotic) endocarditis in the differential diagnosis when evaluating patients with persistent bacteremia and embolic phenomena, as it can mimic infective endocarditis in clinical presentation [6].

Another important consideration is the possible association between *Listeria *infection and high-grade AV block. Although *Listeria *is not a common cause of conduction abnormalities, it has been implicated in cases of endocarditis and myocarditis, leading to conduction disturbances. Reports suggest that *Listeria *may cause direct cardiac involvement, which could contribute to rhythm abnormalities in patients with cardiac implantable electronic devices [7]. Given the rarity but potential severity of this association, clinicians may benefit from a systematic approach: (1) monitor for persistent bacteremia in patients with cardiac devices; (2) assess for new conduction abnormalities; (3) obtain TEE to evaluate for vegetations or valvular involvement; and (4) involve electrophysiology and infectious disease specialists early to guide management and device removal decisions [3].

The patient’s initial gastrointestinal symptoms may have represented an early manifestation of *Listeria* infection, as the bacterium is known to cause gastroenteritis in older adults [8]. The absence of systemic symptoms and omission of blood cultures during that initial hospitalization may have contributed to a delay in diagnosis.

## Conclusions

This case reinforces the importance of early identification and prompt management of *Listeria* infections in high-risk patients. A multidisciplinary approach involving infectious disease specialists, cardiologists, and vascular surgeons was essential for optimizing outcomes in this complex infection involving prosthetic devices and the vascular system. Timely diagnosis and appropriate intervention, including device removal when necessary, were critical in preventing complications and improving the patient’s outcome. The case highlights the multifaceted challenges of diagnosing and managing *L. monocytogenes *infection in elderly patients with cardiovascular comorbidities and implanted prosthetic devices. Although *Listeria* is most commonly associated with sepsis and meningitis, it can rarely cause septic arterial embolism and cardiac device infections. The persistent bacteremia observed in this patient, despite appropriate antibiotic therapy, raised strong suspicion for endovascular infection and ultimately necessitated pacemaker removal. Notably, the potential association between *Listeria* and conduction abnormalities, including high-grade AV block, should be considered when evaluating unexplained cardiac symptoms in at-risk individuals.

This case is unique in demonstrating *Listeria*’s ability to cause persistent bacteremia, septic embolization, and conduction disturbances in the absence of vegetations on imaging and with negative device cultures, thereby expanding the current understanding of *Listeria* pathogenesis in prosthetic device infections. It emphasizes that biofilm-associated infection or endovascular seeding may occur without classic imaging findings, underscoring the need for high clinical suspicion even when echocardiographic and culture results are inconclusive. Early recognition and multidisciplinary coordination were pivotal in the successful management of this case. It underscores the necessity of maintaining a high index of suspicion for device-related infection, even when conventional diagnostic tools are non-revealing. Leadless pacemakers may offer a promising alternative to reduce the risk of recurrent infection in select patients. Overall, prompt diagnosis, targeted antimicrobial therapy, and careful consideration of device extraction remain essential for improving outcomes in patients with invasive *Listeria* infections involving vascular or cardiac structures.
